# Unique colonoscopic manifestations in a patient with Henoch-Schönlein Purpura: a case report of an ileocecal valve ulcer

**DOI:** 10.3389/fped.2025.1728450

**Published:** 2025-12-10

**Authors:** Haizhi Tan, Xiaobing Xiao, Jianrong Deng

**Affiliations:** Department of Pediatrics, Yuebei people’s Hospital, Shaoguan, Guangdong, China

**Keywords:** Henoch-Schönlein Purpura (HSP), IgA vasculitis, ileocecal valve ulcer, abdominal pain, child–Pugh patient

## Abstract

Ileocecal valve ulceration can arise from various causes, including infections, inflammatory bowel diseases, Behçet's disease, tumors and medications. We present a rare case of Henoch-Schönlein purpura (HSP)-associated ileocecal ulcer in a pediatric patient. The child initially presented with abdominal pain and subsequently developed extensive bilateral lower limb purpura on the third hospital day. Colonoscopic examination revealed erosions and shallow ulcerations localized to the ileocecal valve, with histopathological analysis revealing characteristic mucosal stromal edema and diffuse inflammatory cell infiltration. The patient exhibited marked clinical improvement following intravenous methylprednisolone pulse therapy. This case enriches the clinical spectrum of HSP and helps clinicians better understand its intestinal involvement.

## 1 Introduction

The ileocecal region, which begins at the ascending colon and includes the cecum, appendix, and terminal portion of the ileum, has unique anatomical and physiological features such that a wide range of diseases can develop in this area. Ulcerative conditions represent approximately 45%–50% of the diseases affecting the ileocecal region (1). Literature reports show that ileocecal valve ulceration can arise from various causes, including infections (such as tuberculosis and cytomegalovirus), inflammatory bowel diseases (such as ulcerative colitis and Crohn's disease), Behçet's disease, tumors (including cecal cancer, lymphoma, and leiomyoma), and medications (such as hydroxyurea, tacrolimus, and chemotherapy) (1–8). This study reports a rare case of ileocecal ulceration caused by HSP in a pediatric patient.

## 2 Case presentation

A 4-month-old girl was admitted to the pediatric ward of Yuebei People's Hospital in Shaoguan on March 18, 2023, for abdominal pain that had lasted one week. According to the patient's and her family's description, the abdominal pain was predominantly localized in the left periumbilical region, occurring intermittently without a specific temporal pattern, with the nature of the pain remaining poorly characterized. The patient did not experience vomiting, bloody stools, abdominal distension, diarrhea, or fever. A few rashes were observed on the buttocks and thighs, and there was no pain in the joints of the extremities. Before admission, the patient had received glycerin enemas for bowel movement, cefotaxime for infection, and fluid replacement at another hospital, but the therapeutic response remained suboptimal. Consequently, the patient was transferred to our hospital for hospitalization and further treatment. On the third day after admission, the patient suddenly developed numerous purpuric rashes on both lower extremities, accompanied by recurrent abdominal pain. After a glycerin enema, the patient passed through brown, watery stool with debris.

Key laboratory investigations at admission are summarized in Table 1. Notable findings included leukocytosis with neutrophilia, elevated C-reactive protein and D-dimer levels, and abnormal urinalysis showing hematuria and proteinuria. The remaining laboratory test results were as follows: stool examination revealed 1–3 white blood cells per high-power field and occult blood (+). Tests for anti-streptolysin O, rheumatoid factor, IgM antibodies against enterovirus, IgG antibodies against enterovirus, stool culture, serum amylase, serum lipase, troponin, myocardial enzymes, liver and kidney function, coagulation function, electrolytes, and lipid profiles were all normal. The results of the purified protein derivative (PPD) test at 48 h and 72 h were negative.

**Table 1:** Key laboratory findings at admission.

Parameter	Patient's value	Reference range
Complete blood count		
White blood cell count (×10⁹/L)	13.35	4–10
Neutrophils (%)	79.4	50–70
Hemoglobin (g/L)	135	110–150
Platelet count (×10⁹/L)	435	100–300
Inflammatory markers		
C-reactive protein (mg/dL)	18.4	0–0.6
Erythrocyte sedimentation rate (mm/h)	4.0	0–20
Procalcitonin (ng/mL)	0.118	0–0.05
Coagulation profile		
D-dimer (mg/L)	6.9	0–0.55
Renal function & urinalysis		
Blood urea nitrogen (mmol/L)	2.65	2.5–6.5
Creatinine (μmol/L)	26.4	19–44
Urinalysis: Occult blood	2+	–
Urinalysis: Protein	1+	–
Immunological tests		
Serum IgA (g/L)	1.0	1.0–4.2
Antinuclear antibody (U/mL)	6.46	0–18
Complement C3 (g/L)	1.25	0.7–1.4
Complement C4 (g/L)	0.37	0.1–0.4

An abdominal ultrasound performed in our outpatient department prior to admission suggested edema of the transverse and descending colon walls, increasing the possibility of colitis. However, given the patient's persistent and significant abdominal pain and considering that an ultrasound from another hospital prior to transfer had raised the suspicion of intussusception, we proceeded with an abdominal CT scan with contrast agent. The aim was to definitively rule out intussusception or other complications, such as obstruction, and to assess the extent and severity of bowel inflammation more comprehensively. An abdominal CT scan revealed slight swelling of the small intestine, transverse colon, and descending colon walls, with layered enhancement on enhanced scans, suggesting possible inflammation.

**Figure 1:**
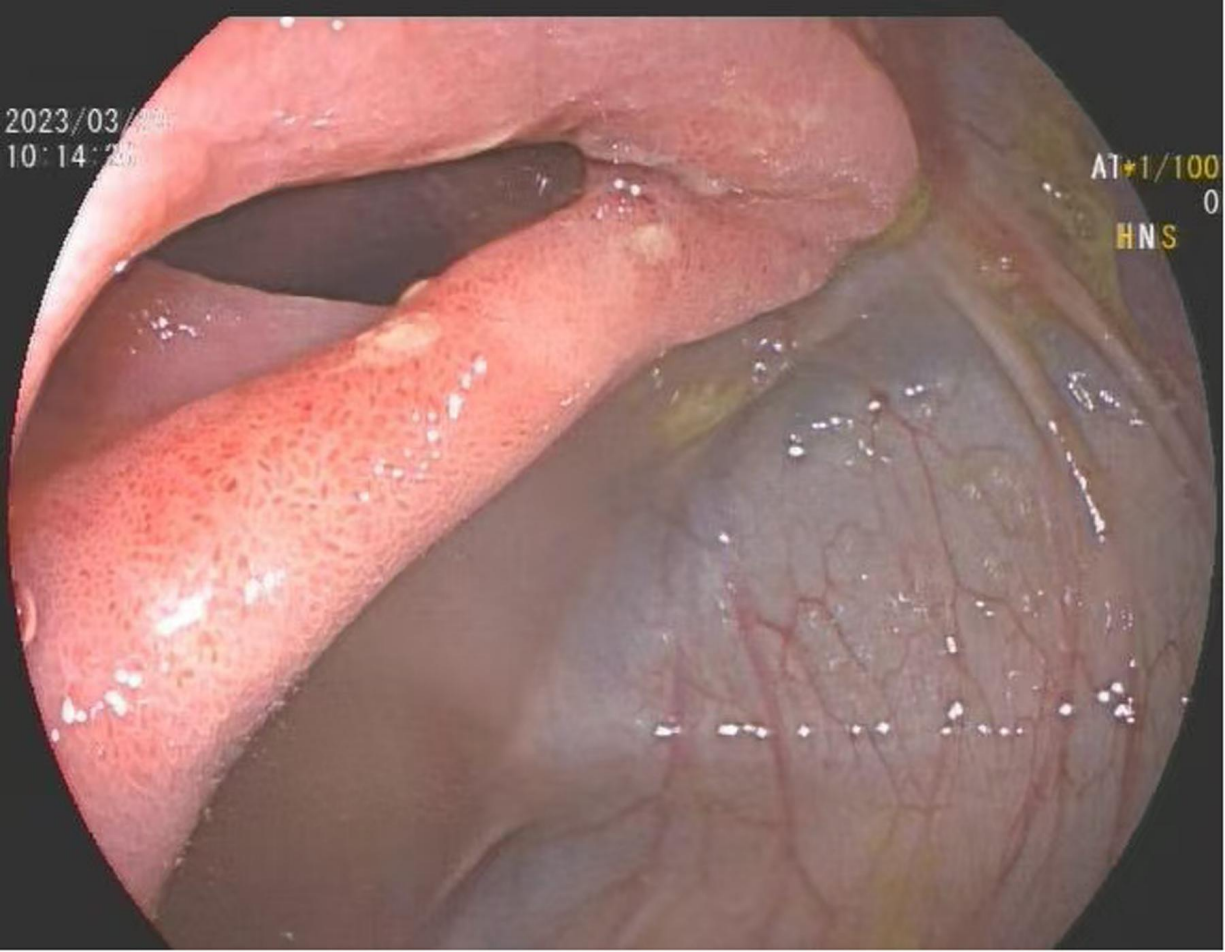
Colonoscopic findings in patients. Colonoscopy revealed congestion and edema of the terminal ileum, with erosion and shallow ulcer formation in the ileocecal valve.

**Figure 2:**
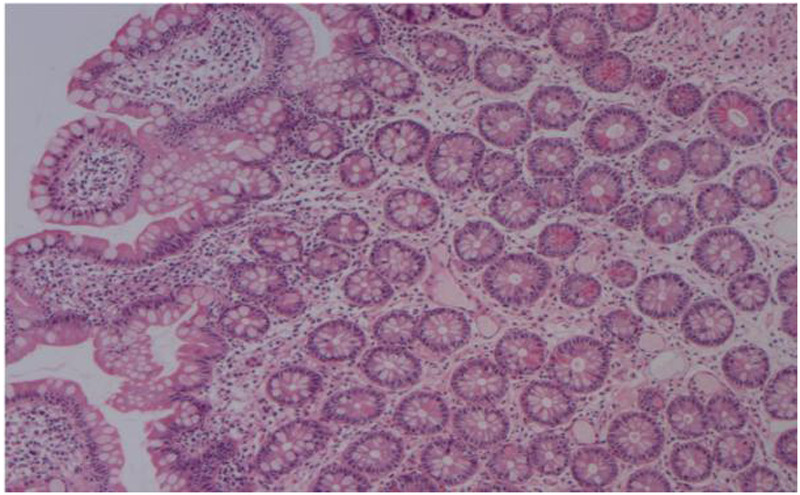
Pathology biopsy findings in patients. The biopsy revealed interstitial edema, dilated small blood vessels, and infiltration of lymphocytes, plasma cells, and neutrophils in the intestinal mucosa. The glandular structure is normal with cryptitis only, indicating mild inflammatory activity (HE staining: 5×).

On the third day after admission, the patient suddenly developed numerous purpuric rashes on both lower extremities, accompanied by recurrent abdominal pain. After a glycerin enema, the patient passed through brown, watery stool with debris. On the basis of the typical purpuric rashes on the patient's lower extremities, which did not fade when pressed, we diagnosed her with mixed-type Henoch–Schönlein purpura. Initially, the patient was treated with intravenous methylprednisolone (2 mg/kg/day) for 2 days. However, the patient's abdominal pain did not significantly improve, and the purpuric rashes continued to progress. Considering the worsening of clinical symptoms, the therapeutic regimen was escalated to intravenous methylprednisolone pulse therapy (20 mg/kg per day) for 3 days. Concurrently, the patient received ceftazidime for anti-infection coverage and low-molecular-weight heparin for anticoagulation. Following the initiation of methylprednisolone pulse therapy, the patient's abdominal pain significantly improved, and the rashes gradually subsided. Gastrointestinal endoscopy was initially used to investigate the cause of persistent abdominal pain and occult gastrointestinal bleeding. However, the procedure was postponed because of inadequate bowel preparation secondary to severe abdominal pain and poor oral intake. After corticosteroid therapy led to significant clinical improvement, a colonoscopy was ultimately performed on the 11th hospital day. This decision was made in consideration of the parents' persistent anxiety and strong request for a definitive endoscopic evaluation and to directly visualize the mucosal condition given the prior severity of symptoms. Endoscopy revealed congestion and edema with scattered erosions predominantly in the gastric antrum and descending segment. Colonoscopy revealed congestion and edema of the terminal ileum, with erosion and shallow ulcer formation in the ileocecal valve (Figure 1). The pathological biopsy revealed interstitial edema in the intestinal mucosa. It also shows dilation of small blood vessels and infiltration of lymphocytes, plasma cells, and neutrophils in the interstitium. The glandular structure appears normal, showing only cryptitis without any crypt abscesses. The absence of definitive epithelioid granulomas was consistent with the mild active mucosal inflammation observed in the mucosa (Figure 2). Following discharge, the patient was maintained on a tapering regimen of oral prednisone. The initial dose was 2 mg/kg per day, which was reduced by 0.5 mg/kg per day on a weekly basis until discontinuation, resulting in a total corticosteroid course of 4 weeks. A follow-up colonoscopy at 3 months was recommended to monitor ulcer healing but was declined by the parents due to complete symptom resolution and reluctance toward repeat invasive procedures. Clinical surveillance was therefore maintained, and the patient remained asymptomatic without recurrence of abdominal pain or purpura throughout the 3-month follow-up, indicating sustained clinical remission.

## 3 Discussion

This study reports a rare pediatric case of ileocecal valve ulceration caused by HSP. HSP is an IgA-mediated systemic small-vessel vasculitis predominantly affecting the skin, gastrointestinal tract, kidneys and joints. It is the most common form of systemic vasculitis in children. Global epidemiological studies indicate that HSP, as the most prevalent systemic vasculitis in children, has an incidence rate of 10–20 cases per 100,000 children, with approximately 90% of cases occurring between the ages of 2–10 years and exhibiting incidence peaks at 4–7 years (9). Gastrointestinal manifestations occur in 50%–80% of pediatric HSP patients, primarily presenting as abdominal pain, vomiting, diarrhea, and hematochezia, and a small proportion may develop severe complications, including intussusception, intestinal perforation, or bowel obstruction (10). Approximately 10%–40% of HSP patients present with gastrointestinal symptoms as their initial manifestation (11).

The mucosa of the entire gastrointestinal tract, including the esophagus, stomach, duodenum, jejunum, ileum, and colon, can be involved in patients with HSP (12). A study of adult HSP patients with abdominal symptoms revealed that 87% of endoscopic examinations revealed abnormalities, primarily mucosal ulcers (13). Studies have also shown that the most common endoscopic finding in the upper gastrointestinal tract is tract erythema/petechiae, whereas the most frequent findings in the lower gastrointestinal tract are erosions/ulcers (14). In patients with HSP, duodenal involvement, especially in the descending segment, is most commonly observed during gastroscopy (15–17). During colonoscopy, the terminal ileum is often the most severely affected site (17). Diffuse erythematous hyperemia, mucosal eruptions, erosions, and superficial multiple ulcers, particularly in the duodenal descending segment or terminal ileum, are characteristic endoscopic findings of HSP (12, 17, 18). Ileocecal valve ulceration is relatively rare in patients with HSP. Currently, we know of no publications on this topic. Therefore, the findings of this case enrich the clinical spectrum of HSP and help clinicians better understand its intestinal involvement. The differential diagnosis for ileocecal ulceration is broad and includes infectious, inflammatory, autoimmune, and neoplastic causes. Infectious causes such as intestinal tuberculosis were deemed unlikely given the patient's negative purified protein derivative test, absence of constitutional symptoms (e.g., fever, night sweats), and lack of caseous granulomas or necrosis on histopathology (19).

Inflammatory bowel disease (IBD), particularly Crohn's disease, was considered; however, the acute presentation, the characteristic evolution of purpuric rash, the absence of chronic diarrhea or weight loss, and the biopsy findings showing cryptitis without noncaseating granulomas or focal crypt architectural abnormalities contradict this diagnosis (20). Although Behçet's disease can cause ileocecal valve ulcers, it typically presents with large ulcers (>2 cm) or circumferential ulcers and may be accompanied by recurrent oral aphthae, genital ulcers, or ocular lesions (21, 22). Furthermore, drug-induced ulcers were considered improbable, as there was no history of chronic medication use (e.g., NSAIDs, hydroxyurea) known to cause such lesions. The temporal sequence of abdominal pain preceding classic nonblanching purpura, combined with the characteristic endoscopic findings and the exclusion of these alternative conditions, strongly supported the diagnosis of HSP as the cause of ileocecal valve ulceration.

The treatment for this patient included anti-infective therapy, corticosteroid pulse therapy, and anticoagulation. This approach significantly improved the patient's symptoms and gradually resolved the rash. After discharge, the patient continued oral prednisone therapy for consolidation and remained free of recurrence during the 3-month follow-up period. Importantly, there are no standardized or evidence-based treatment guidelines specifically for HSP-associated ileocecal valve ulceration, given its rarity. Our therapeutic approach was therefore extrapolated from general principles for managing severe gastrointestinal involvement in HSP, which recommend corticosteroids for significant symptoms such as severe abdominal pain and bleeding.

According to the SHARE Initiative (23) and the Chinese Guidelines (24) for Pediatric IgA Vasculitis, the routine treatment regimens for HSP include the following: the use of corticosteroids for significant abdominal pain or bleeding; the use of oral prednisolone at a dose of 1–2 mg/(kg·d) is commonly used in clinical practice; and for patients with severe symptoms, poor treatment response, or life-threatening manifestations, intravenous pulsed methylprednisolone therapy [10–30 mg/(kg·d) for 3 days] is a recognized escalated treatment strategy. The patient in this case first received pulsed methylprednisolone therapy (20 mg/kg for 3 consecutive days), followed by sequential oral prednisone, which is consistent with the aforementioned treatment principles for severe gastrointestinal involvement in HSP. Immunosuppressants such as azathioprine and mycophenolate mofetil are typically reserved for severe cases or those refractory to corticosteroids, especially when combined with renal involvement. However, the patient in this case achieved rapid and sustained clinical remission with corticosteroid monotherapy, thus eliminating the need for additional immunosuppressive agents. Supportive care, including fluid replacement, analgesia, and close monitoring for surgical complications, is a fundamental component of HSP management and plays a crucial role in the treatment of this patient.

These findings suggest that timely and effective treatment may improve the prognosis of children with HSP who have intestinal involvement. However, a limitation of this case was the parents' reluctance to undergo gastrointestinal endoscopy, which prevented follow-up endoscopy during treatment. In conclusion, this case underscores that Henoch–Schönlein purpura can present with discrete ulceration at the ileocecal valve, a rarely reported site. These findings highlight the diagnostic value of endoscopy in children with unexplained abdominal pain and support the use of corticosteroid pulse therapy for severe HSP with significant ulcerative gastrointestinal lesions. This report aims to increase clinical vigilance for this manifestation to facilitate timely diagnosis and management.

## 4 Clinical significance

This case underscores that Henoch-Schönlein purpura can manifest with discrete ulceration at the ileocecal valve, a rarely reported site, thereby enriching the known clinical spectrum of its gastrointestinal involvement. This highlights the critical diagnostic value of colonoscopy in children presenting with unexplained abdominal pain, as direct visualization can confirm vasculitic lesions and rule out other pathologies. Furthermore, our experience supports the efficacy of intravenous methylprednisolone pulse therapy, followed by an oral taper, in achieving rapid remission of severe ulcerative gastrointestinal lesions in HSP, offering a valuable therapeutic reference for managing similar complex cases.

## Data Availability

The raw data supporting the conclusions of this article will be made available by the authors, without undue reservation.
